# Prevalence of heteroploidy in plastic film-induced primary sarcomas.

**DOI:** 10.1038/bjc.1966.69

**Published:** 1966-09

**Authors:** M. R. Banerjee, R. R. Bates

## Abstract

**Images:**


### PREVALENCE OF HETEROPLOIDY IN PLASTIC FILM-INDUCED PRIMARY SARCOMAS

M. R. BANERJEE AND RICHARD R. BATES

*From the Biology Branch, Carcinogenesis, Etiology, National Cancer Institute, Bethesda, Maryland, U.S.A.*

Received for publication April 28, 1966

DURING the past several years critical facts have been collected about the chromosomes of neoplastic cells, mostly using ascites and transplanted tumors. A vast majority of these tumors was found to be associated with various chromosomal abnormalities and several investigators suggested that karyotypic alterations are of primary significance to the onset of neoplasia (Makino, 1957 ; Ford, Hamerton and Mole, 1958 ; Levan, 1959). On the other hand, chromosome study of the primary tumors revealed that a large number of these tumors was predominantly diploid (Bayreuther, 1960) and in addition, comparison of the chromosome constitiution of some primary mouse tumors with that of the immediately preneoplastic tissue (Blair, DeOme and Nandi, 1962), from which the tumors were derived, also failed to demonstrate any correlation (Banerjee and DeOme, 1963). On the basis of the latter findings, it is becoming more and more apparent that deviations from the normal karyotype in tumor cells are probably associated with tumor progression rather than causation of neoplasia. Evidence for the above views has been discussed by several authors (Levan, 1959 ; Bayreuther, 1960 ; Hauschka, 1961).

Most experimental tumors whose karyotype has been studied so far were either radiation, chemical, or virus-induced and all these carcinogenic agents have been shown to be capable of producing alterations in chromosome number and form (Ford and Mole, 1959 ; Hellström, 1959 ; Hellström, Hellström and Sjogren, 1962). With the discovery that plastic, a rather inert material, could act as a carcinogenic agent (Oppenheimer, Oppenheimer and Stout, 1952), several investigators became interested in this intriguing tumorigenic process (Goldhaber, 1961 ; Bates and Prehn, 1965). In this situation, neoplastic change is induced by a substance that virtually has no chemical activity or toxic properties (Oppenheimer *et al*, 1955 ; Vasiliev *et al*, 1962).

In view of the above findings, a study of the karyotype of tumors induced by plastic appeared to be of much interest and the present paper reports the chromosome constitution of 20 such tumors.

### MATERIALS AND METHODS

BALB/c mice of both sexes, 1–3 months old, obtained either from the main National Institutes of Health breeding colony or Cumberland View Farms, were used. Polyethylene discs, 0·015 inches in thickness and 2·0 centimeters in diameter were implanted underneath the skin on the left side of the backs of the mice.

A small incision in the skin at the site of the implanation was made under ether anesthesia and each disc inserted through the slit-like opening. Before insertion, the discs were treated in 70% ethyl alcohol followed by rinsing in 0·85% sterile saline. The opening in the skin was closed by wound clips and in some cases, for younger animals, 3·5% celloidin was used.

The first tumors began to appear at the site of the implant 7 months after the operation. Animals were killed by cervical dislocation and tumors of varying sizes were collected, one tumor per mouse. One hour before killing the animals received 0·2 ml. of 0·5% colchicine by intraperitoneal injection. Following the removal of the tumors, their length, width and depth were measured in millimeters. The product of the 3 dimensions of each tumor has been expressed as the " relative size " (Table I). The plastic discs from all the tumors were recovered intact. Part of the tumor tissue was fixed for histologic examination and the remaining portion used for chromosome analysis. Tumor tissues fixed for histologic study were prepared according to the usual paraffin-embedding schedule and the sections were stained with hematoxylin and eosin. Portions of tumor saved for chromosome preparation were minced into small cubes and treated in distilled water for 15–20 minutes. After the hypotonic treatment, tissue cubes were fixed in 50% acetic acid, squashed and stained with aceto-orcein. Samples of spleen tissue obtained from the tumor bearing animals, also handled in the same way, were used for chromosome analysis of the non-tumor tissue.

Tumor bearing mice used in this study were part of another experiment by one of the authors and these results are published in a separate paper (Bates and Klein, 1966).

### RESULTS

A total of 26 tumors was collected and 20 were examined for chromosome analysis while the remaining 6 were discarded because of the lack of adequate number of metaphase figures. The neoplastic nature of each tumor was determined by histologic examination. All the tumors were sarcomas, composed primarily of spindle shaped cells. The more pleomorphic tumors contained large cells with big hyperchromatic nuclei and many of the latter cells also had multiple nuclei. The amount of collagen found within the tumor tissues varied from being scant in some cases to abundant in others. Table I summarizes the data on the relative size, histologic characteristics and the distribution of chromosome number in the different tumors.

All the sarcomas examined during this study were highly heteroploid and the frequency of cells with different chromosome numbers was distributed over a wide range, extending from hypodiploid to hypertetraploid. Although heteroploidy was the general feature in almost all of these sarcomas, in some cases an apparent modal chromosome number was also observed. Tumors 12 and 17 contained a large number of cells with 40 chromosomes (63% and 58%, respectively with relatively few heteroploid cells. A slightly above diploid modal chromosome number was observed in tumor 6, where 51% of the cells had 43 chromosomes. Tumors 4 and 8 contained a predominantly high number of hypertetraploid cells but in both these tumors, the number of chromosomes in many of the hypertetraploid cells was so high that an exact count was difficult. Besides the high chromosome number, both these tumors also contained two distinct types of structural abnormalities of the chromosomes. Variable numbers of minute chromosomes (m-chromosome), a negatively heteropycnotic metacentric element, or both, were present in 50% of the cells in tumor number 4 (Fig. 1). Structurally

TABLE I.—*Relative Size, Histologic Characteristics and Distribution of Chromosome Numbers in 20 Mouse Sarcomas Induced by Plastic Film Implantation.*

| Tumor No. | *Relative Size | †Degree of pleomorphism | Chromosome number (frequency) | | | | | | | | | | | | | | | | | | | | |
|---|---|---|---|---|---|---|---|---|---|---|---|---|---|---|---|---|---|---|---|---|---|---|---|
| | | | 30–35 | 36 | 37 | 38 | 39 | 40 | 41 | 42 | 43 | 44 | 45 | 46 | 47 | 48 | 49 | 50–60 | 61–70 | 71–79 | 80 | 80> | Tot. |
| 1 | 64 | ++++ | .. | .. | .. | .. | .. | 4 | .. | .. | .. | .. | .. | .. | .. | .. | .. | .. | .. | .. | .. | .. | 4 |
| 2 | 108 | ++++ | 4 | 1 | 1 | .. | .. | 13 | .. | .. | 1 | .. | .. | 1 | 1 | .. | 1 | 2 | .. | 1 | 4 | 9 | 39 |
| 3 | 126 | ++++ | 1 | .. | .. | .. | .. | 4 | .. | .. | 2 | .. | 8 | 2 | 1 | .. | .. | 1 | .. | .. | .. | 1 | 20 |
| 4 | 168 | ++++ | .. | 2 | .. | .. | .. | 2 | .. | .. | 1 | .. | .. | .. | 1 | .. | 1 | 4 | 1 | 1 | .. | 19 | 32 |
| 5 | 210 | +++ | .. | .. | .. | .. | .. | 6 | 2 | .. | 1 | .. | 4 | 1 | .. | 1 | .. | 13 | 1 | 1 | 4 | .. | 34 |
| 6 | 252 | ++ | .. | .. | 1 | 1 | .. | 1 | .. | 4 | 21 | 2 | 5 | 2 | 1 | 1 | 1 | .. | 1 | .. | .. | .. | 41 |
| 7 | 280 | ++ | .. | .. | .. | 1 | 2 | 5 | .. | .. | .. | .. | .. | .. | 1 | .. | 1 | 5 | 3 | 7 | .. | 1 | 26 |
| 8 | 540 | ++ | .. | .. | .. | 1 | 1 | 8 | .. | .. | 1 | .. | 1 | .. | .. | .. | .. | 2 | 3 | 5 | 4 | 17 | 43 |
| 9 | 608 | ++ | 3 | 1 | 1 | 2 | 2 | 4 | 15 | 14 | 5 | 1 | 2 | .. | .. | .. | .. | .. | .. | .. | 1 | .. | 51 |
| 10 | 693 | ++++ | .. | .. | 2 | .. | 1 | 3 | 2 | 2 | .. | 1 | .. | 2 | .. | .. | .. | .. | .. | 9 | 4 | .. | 26 |
| 11 | 864 | ++ | 6 | 1 | .. | .. | .. | 5 | 1 | 2 | 1 | 2 | 2 | 5 | 5 | 6 | 4 | 2 | .. | 1 | .. | .. | 43 |
| 12 | 968 | ++ | 9 | .. | 2 | .. | .. | 24 | .. | .. | 1 | .. | 1 | .. | .. | .. | .. | .. | .. | .. | .. | 1 | 38 |
| 13 | 1008 | ++ | 5 | 1 | 3 | 2 | 1 | 1 | 1 | 1 | 6 | 2 | 1 | .. | .. | .. | .. | .. | 1 | .. | .. | 2 | 27 |
| 14 | 1014 | ++ | .. | .. | .. | .. | .. | 2 | 3 | 1 | .. | .. | .. | .. | .. | .. | .. | .. | .. | .. | .. | .. | 6 |
| 15 | 1134 | +++ | .. | .. | 1 | .. | 1 | 2 | .. | .. | .. | 1 | .. | .. | .. | 1 | .. | 4 | 9 | 5 | 3 | 1 | 28 |
| 16 | 1760 | + | .. | .. | 2 | .. | .. | 2 | 6 | 9 | 3 | 11 | 5 | 1 | .. | 1 | 1 | 2 | .. | 1 | .. | 1 | 45 |
| 17 | 2016 | + | 3 | 1 | .. | .. | 2 | 14 | 1 | 2 | .. | .. | .. | .. | .. | .. | .. | .. | .. | .. | 1 | .. | 24 |
| 18 | 2100 | ++ | 4 | 1 | 1 | .. | .. | 8 | 1 | 4 | 4 | 1 | .. | 1 | .. | .. | .. | .. | .. | .. | .. | 1 | 26 |
| 19 | 5460 | ++ | 3 | 1 | 1 | 1 | 1 | 10 | 6 | 3 | 4 | .. | .. | 1 | .. | .. | .. | .. | .. | .. | .. | 1 | 32 |
| 20 | 5720 | ++ | 2 | .. | 2 | 1 | 5 | 1 | 7 | 5 | 8 | 6 | .. | 3 | 2 | 1 | .. | .. | 1 | .. | .. | 1 | 45 |
| Spleen (control) | | | .. | .. | .. | .. | 1 | 39 | .. | .. | .. | .. | .. | .. | .. | .. | .. | .. | .. | .. | .. | .. | 40 |

* Relative size was obtained by multiplying the length of the 3 dimensions of each tumor (see Materials and Methods.)

† Degree of pleomorphism : increasing number of + indicates higher degree of pleomorphism.

aberrant chromosomes in a high frequency of cells were also oebsrved in 6 other tumors. A distinct sub-metacentric chromosome, in some cases accompanied by m-chromosomes, was present in approximately 50% of the cells in tumor 3 (Fig. 2) and 58% of the cells in sarcoma 9 were characterized by an unpaired, large telocentric element (Fig. 3). In addition to the above tumors, m-chromosomes were also observed in an appreciable number of cells in tumors 8, 10,18 and 20.

Histologically there was a considerable amount of heterogeneity among the 20 tumors particularly with regard to their degree of pleomorphism. However, an interesting relationship between relative size of the sarcomas and the degree of pleomorphism was quite clearly visible (Table I). Relatively small tumors were noticeably more pleomorphic as compared to the large sarcomas (Fig. 4 and 5).

In view of the above relationship, the sarcomas were arbitrarily divided into two size groups, those with a relative size of 800 or less, as small, and tumors with a relative size of more than 800 were considered as large. Data on the chromosome number of the tumors of each size group were pooled and plotted on two separate histograms (Fig. 6 and 7). Tumors of the small, relative-size group showed two distinct peaks, one at the diploid and near diploid chromosome numbers and another at tetraploid and above tetraploid region. Besides, in this group 39% of the cells contained chromosome numbers ranging from 50 to tetraploid and above tetraploid whereas in the tumors of the large size group the same range comprised only 12% of the cells. Furthermore, the small tumors also contained a wide spectrum of intermediate variables with chromosome numbers between 50 and 79 and the same spectrum of intermediate variables (50 to 79) was noticeably decreased in the large tumors (Fig. 6 and 7). However, in the tumors of the large size group, while the peaks at the tetraploid and above tetraploid regions were greatly reduced, the peaks at the diploid and near diploid regions remained distinct and well maintained (Fig. 6 and 7).

Almost all the cells of the splenic tissues contained the diploid number of 40 chromosomes (Table I) and no detectable structural abnormality of the chromosomes was observed. Attempts were also made to examine the karyotype of the capsules formed around the plastic discs. Owing to an extremely inadequate number of dividing cells in this tissue no meaningful evaluation of the data was possible.

### EXPLANATION OF PLATES

FIG. 1.—A hypertetraploid cell from tumor number 4 with 136 chromosomes plus 7 m-chromosomes (arrow). Note the large negatively heteropycnotic metacentric element (arrow) × 580.

FIG. 2.—An above diploid cell from tumor number 3, with 47 chromosomes. Arrow indicates the sub-metacentric element. × 580.

FIG. 3.—A pseudodiploid cell from tumor number 9. Arrow shows the large unpaired telocentric element . × 580.

FIG. 4.—Representative field from a tumor of small relative size group. Note the highly pleomorphic nature of the tissue. × 240.

FIG. 5.—Representative field from a tumor of the large relative size group. Note the well reduced pleomorphic nature of the tissue. × 240.

1

2

3

Bannerjee and Bates.

4

5

### DISCUSSION

The findings of the present study show that the chromosome constitution of plastic film induced primary sarcomas in mice are as variable as many other murine primary tumors (Hauschka, 1961). Although diploid and slightly above diploid modal chromosome numbers were seen in some tumors, in all instances the stem-line chromosome number was obscured by the presence of a wide range of variables.

Since these tumors are induced by materials which are essentially chemically inert (Oppenheimer, *et al*, 1952 ; Vasiliev *et al*, 1962), alterations in chromosome number and structure in the plastic induced sarcoma cells deserve some attention with regard to a possible mechanism for the origin of such karyotypic abnormalities. Inadequate supply of oxygen and nutrients, constant contact of the cells with a foreign object and isolation of the cells from humoral growth control by the host have been suggested to be the important changes that occur in the tissue (Vasiliev *et al*, 1962 ; Alexander and Horning, 1959). The above environmental changes make the situation somewhat comparable to a tissue culture system. In tissue culture of mouse embryonic cells, Levan and Biesele observed that a wide variety of karyotypic abnormalities occur during early stages of the culture and subsequently an adaptive stem-line karyotype may evolve (Levan and Biesele, 1958). Two main selective pressures—altered nutrition and absence of host factors—probably underlie these karyotypic readjustments. In addition, an altered oxygen supply itself has been reported to cause a derangement of the mitotic mechanism and this may lead to various chromosomal changes in the daughter cells (Gavaudan, 1956). Since the sarcomas apparently arise in an area of hyalinized avascular fibrous tissue (Oppenheimer *et al*, 1958), the wide variety of chromosomal rearrangements observed during this study may not be unexpected. Several processes that may lead to chromosomal aberrations are known, e.g., endomitosis, non-disjunction and chromatid breaks (Levan, 1956). In addition to the heteroploid chromosome numbers, minute chromosomes (m-chromosomes), sub-metacentric and metacentric chromosomes observed during the present study indicate the occurrence of chromosome breaks and rearrangements.

A casual relationship between the chromosomal abnormalities and formation of tumors is uncertain. The inconsistency of the karyotype of the different tumors together with the absence of a well defined stem-line in any individual neoplasm tend to indicate that karyotypic alterations and the formation of tumors are two independent events. This view finds support in the observation that chromosomal changes produced by ionizing radiation may persist in man and mice for long periods without the development of tumors (Nowell, Hungerford and Cole, 1964). However, the wide range of heteroploid variations could provide a selective advantage to the tumor cells in a changing environment. The high degree of pleomorphism observed in the small tumors, followed by the noticeably low pleomorphic nature of the large tumors, suggests a change in the tumor environment reaching towards stability. Although the chromosomal constitution of individual sarcomas was inconsistent, the pooled data of the tumors of the small and large size groups revealed some interesting patterns. In addition to the large fraction of cells with diploid and near diploid chromosome numbers, the tumors of the small size group also contained an accumulation of cells with hypertetraploid chromosome number along with a high frequency of cells spread over a wide range of intermediate variable chromosome numbers (Fig. 6). The

FIG. 6.—Distribution of chromosome number in 10 primary sarcomas belonging to the small relative size group ( < 800). For further details see text (Results).

FIG. 7.—Distribution of chromosome number in 10 primary sarcomas belonging to the large relative size group ( > 800). See text for further details (Results).

occurrence of this spectrum of variants clearly demonstrates the cytogenetic heterogeneity of these tumors. Observation of the random, structural aberrations of the chromosomes in 3 out of 10 tumors of this group ; also the high degree of pleomorphism further tends to indicate the unstable state of the tumor tissue during early stages of the neoplastic growth. On the other hand, the karyotype of the larger tumors present a rather less variable nature. There is a drastic reduction in the frequency of cells with hypertetraploid chromosome number and also the number and range of intermediate variants are considerably decreased. However, the persistent occurrence of the peak at the diploid and near diploid region (Fig. 7) tends to suggest the selection of those cells favored by the environment of the tumor tissue. Presence of an apparent modal diploid chromosome number in tumors 12 and 17 of the large size group also lends support to the view relative to the presence of a selective pressure during tumor progression and establishment of comparatively stable karyotypic stem-line. Noticeably low pleomorphic nature of the larger tumors could also be considered as an indication of a relatively stable state of the environment of the sarcomas.

While the primary significance of gross chromosomal mutation in neoplasia remains an open question, findings in this study seem to indicate that karyotypic reorganization, hence new genetic constitution, in neoplastic cells could provide a selective advantage during tumor progression.

### SUMMARY

Chromosome constitution of primary sarcomas of different sizes induced by plastic film was analyzed. In general, the 20 tumors studied were highly heteroploid, and chromosome numbers were distributed over a wide range extending from hypodiploid to hypertetraploid cells. Apparent diploid or near diploid modal chromosome numbers were observed in occasional tumors. In addition to numerical variations, structural aberrations of the chromosomes were also quite frequent in many tumors. Histologically, the sarcomas were highly heteromorphic, relatively small tumors being more pleomorphic than the larger neoplasm. Comparison of the pooled data on the chromosome constitution of relatively small and relatively large tumors revealed that the high degree of variability in the former was noticeably decreased in the latter. There appeared to be a tendency toward establishment of a stem-line(s) in the large tumors. Significance of karyotypic abnormalities in the sarcoma cells providing a selective advantage during tumor progression is discussed.

The authors wish to express their thanks to Dr. Michael Klein for his support, also to Mrs. Ruby Walker and Mr. William Neubauer for their assistance.

### REFERENCES

ALEXANDER, P. AND HORNING, E.—(1959) Ciba Foundation Symposium on Carcinogenesis. London (Churchill).

BANERJEE, M. R. AND DEOME, K. B.—(1963) *Cancer Res.*, **23**, 546

BATES, R. R. AND KLEIN, M. (1966) *J. natn. Cancer Inst.*, **37**, 145.

BATES, R. R. AND PREHN, R. T.—(1965) *Nature, Lond.*, **205**, 303.

BAYREUTHER, K.—(1960) *Nature, Lond.*, **186**, 6.

BLAIR, P. B., DEOME, K. B. AND NANDI, S.—(1962) Henry Ford Hospital International Symposium : Biological Interaction in Normal and Neoplastic Growth. pp. 371–389, Boston (Little Brown and Co.).

FORD, C.E., HAMMERTON, J. L. AND MOLE, R. H.—(1958) *J. cell comp. Physiol.*, **52**, (Suppl. 1), 235.
FORD, C. E. AND MOLE, R. H.—(1959) ' Progress in Nuclear Energy ', Vol. 2, p. 11, New York (Pergamon Press).
GAVAUDAN, P.—(1956) ' Facteurs de la croissance cellulaire ', p. 275, Paris (Masson).
GOLDHABER, P.—(1961) *Proc. Am. Ass. Cancer Res.*, **3**, 228.
HAUSCHKA, T. S.—(1961) *Cancer Res.*, **21**, 957.
HELLSTRÖM, K. E.—(1959) *J. natn. Cancer Inst.*, **23**, 1019.
HELLSTRÖM, I., HELLSTRÖM, K. E. AND SJOGREN, H. O.—(1962) *Exp. Cell Res.*, **26**, 434.
LEVAN, A.—(1956) *Ann N. Y. Acad. Sci.*, **63**, 774.
LEVAN, A.—(1959) ' Genetics and Cancer '. Texas (Austin Univ. Press).
LEVAN, A. AND BIESELE, J. J.—(1958) *Ann. N. Y. Acad. Sci.* **71**, 1022.
MAKINO, S.—(1957) *Int. Rev. Cytol.*, **6**, 25.
NOWELL, P. C., HUNGERFORD, D. A. AND COLE, L. J.—(1964) *Ann. N. Y. Acad. Sci.* **114**, 252.
OPPENHEIMER, B. S., OPPENHEIMER, E. T. AND STOUT, A. P.—(1952) *Proc. Soc. exp. Biol. Med.* **79**, 366.
OPPENHEIMER, B. S., OPPENHEIMER, E. T., STOUT, A. P. AND EIRICH, F. R.—(1955) *Cancer Res.*, **15**, 333.
OPPENHEIMER, B. S., OPPENHEIMER, E. T., STOUT, A. P., WILLHITE, M. AND DANISHEFSKY, I.—(1958) *Cancer, N.Y.* **11**, 204.
VASILIEV, J. M., OLSHEVSKAYA, L. V., RAIKHLIN, N. T. AND IVANOVA, O. J.—(1962) *J. natn. Cancer Inst.*, **28**, 515.


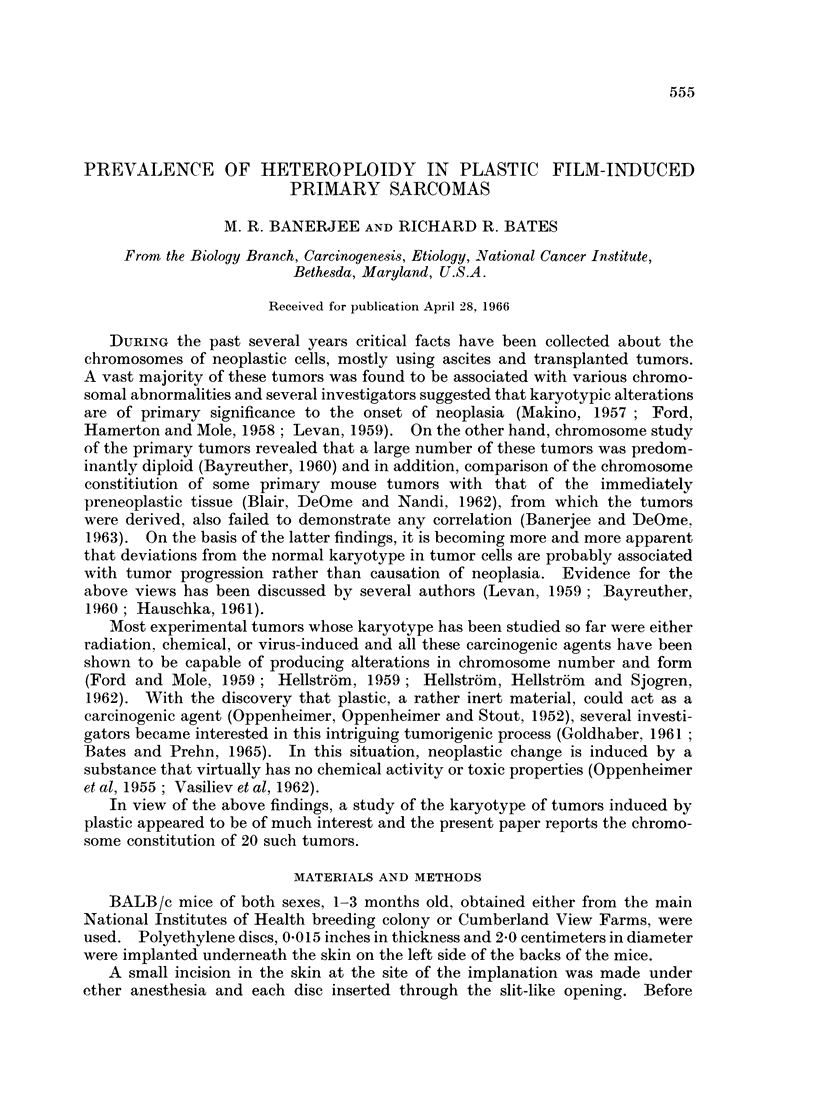

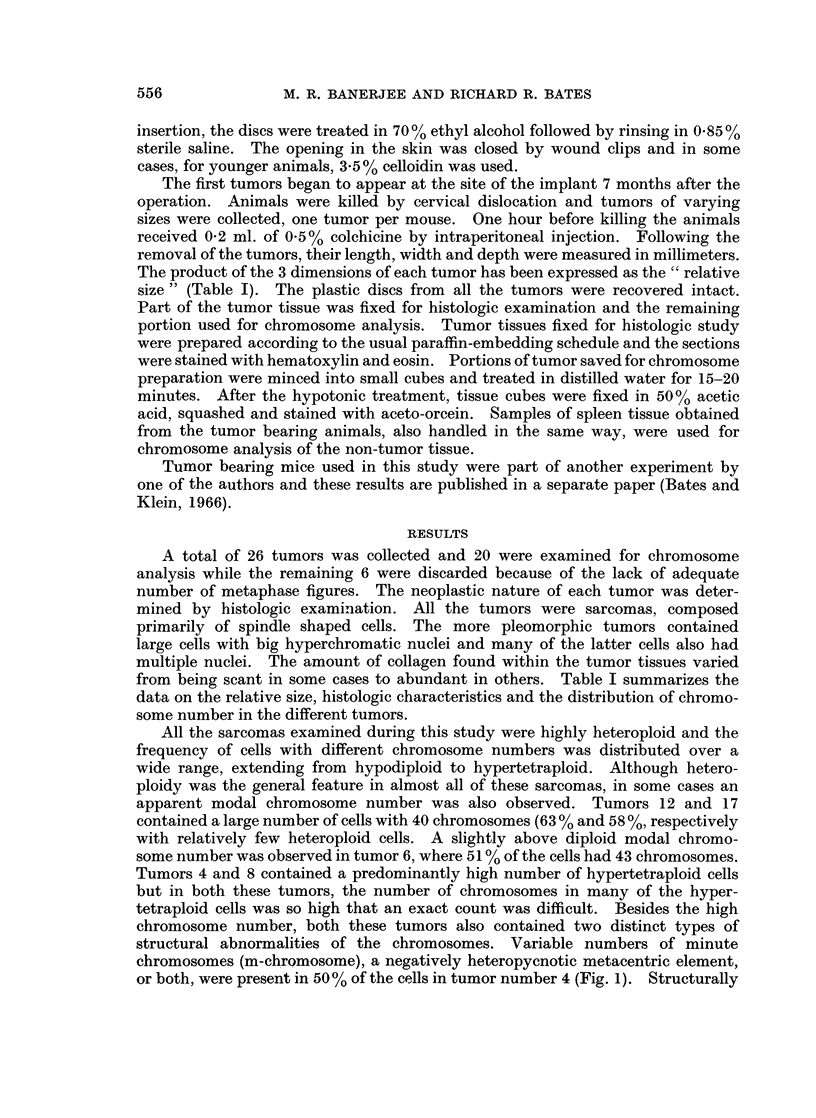

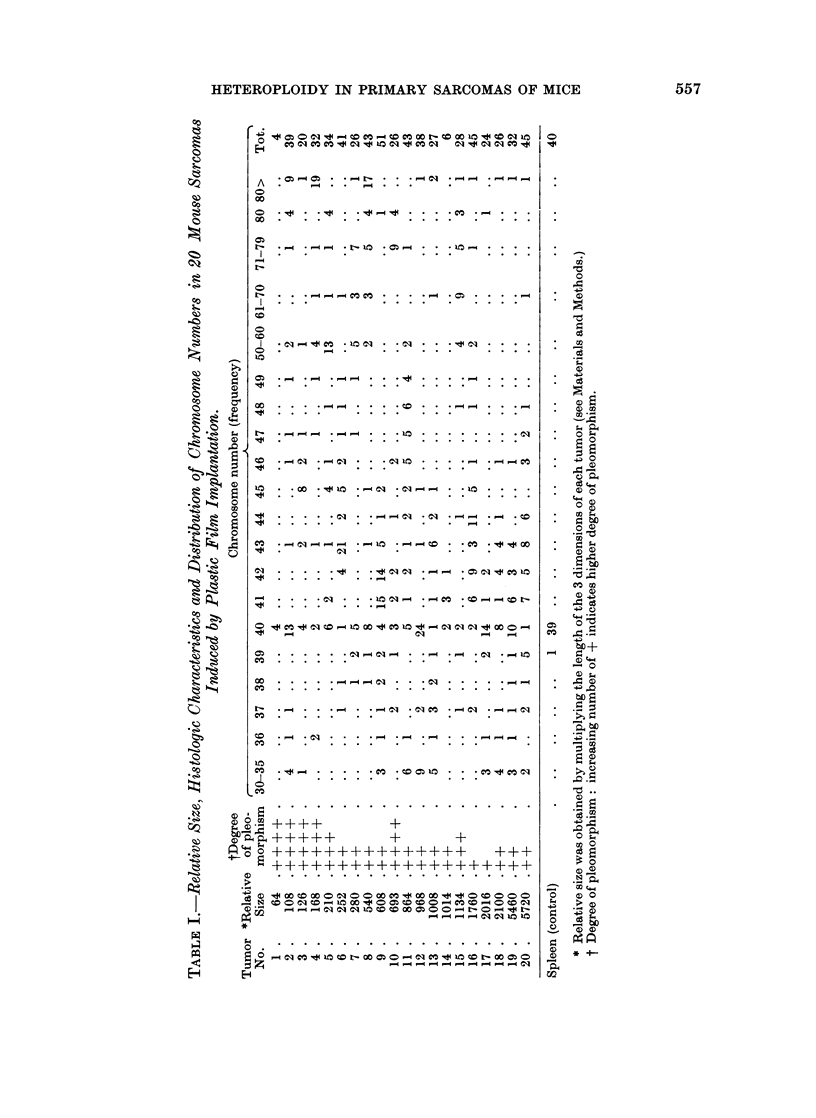

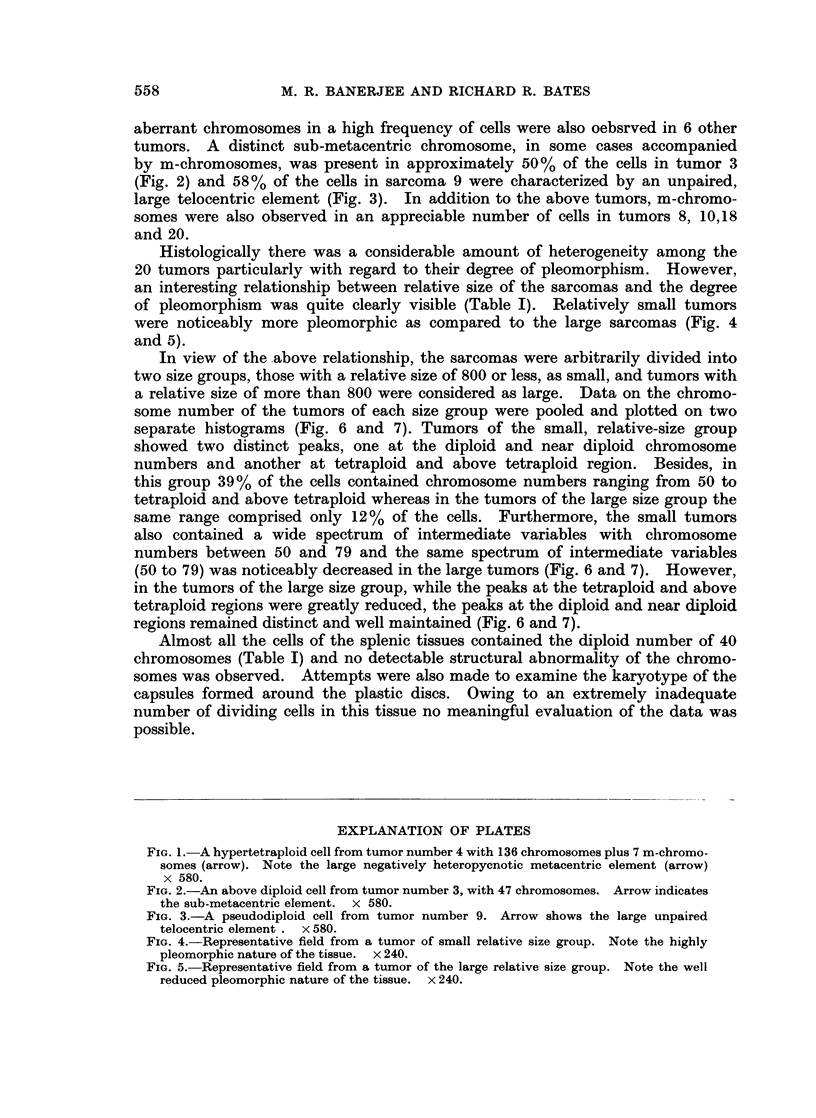

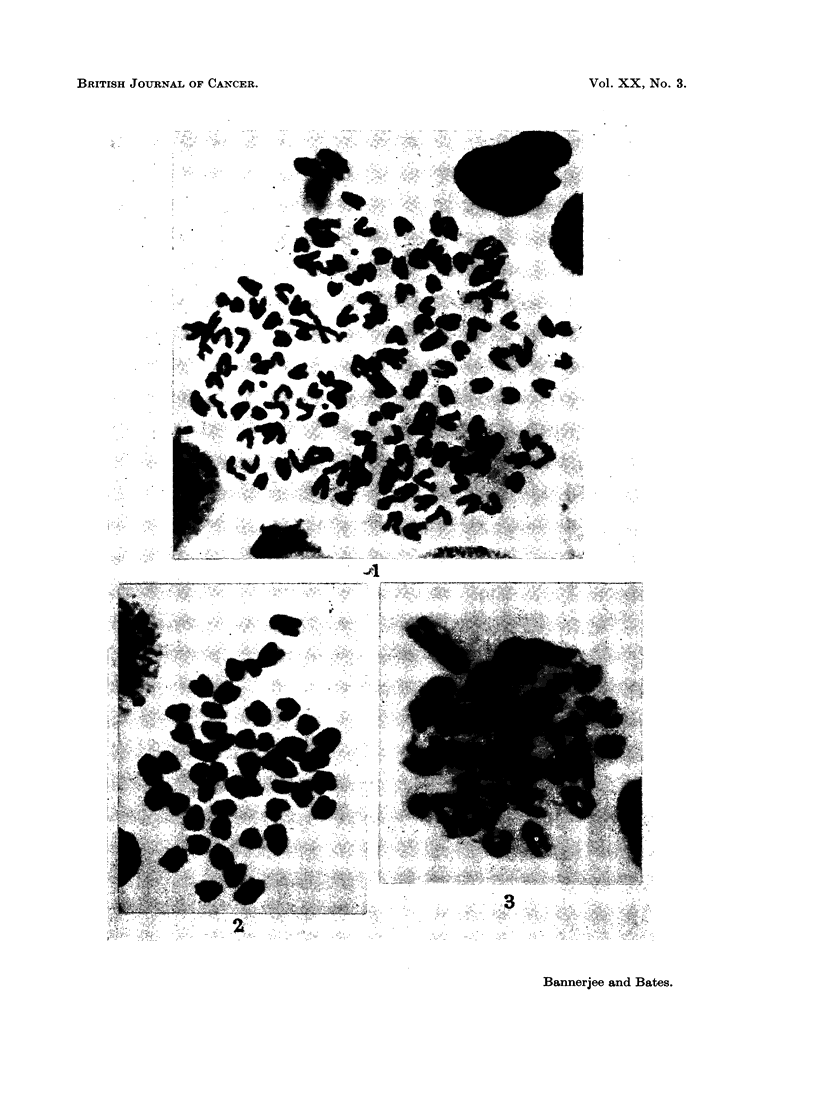

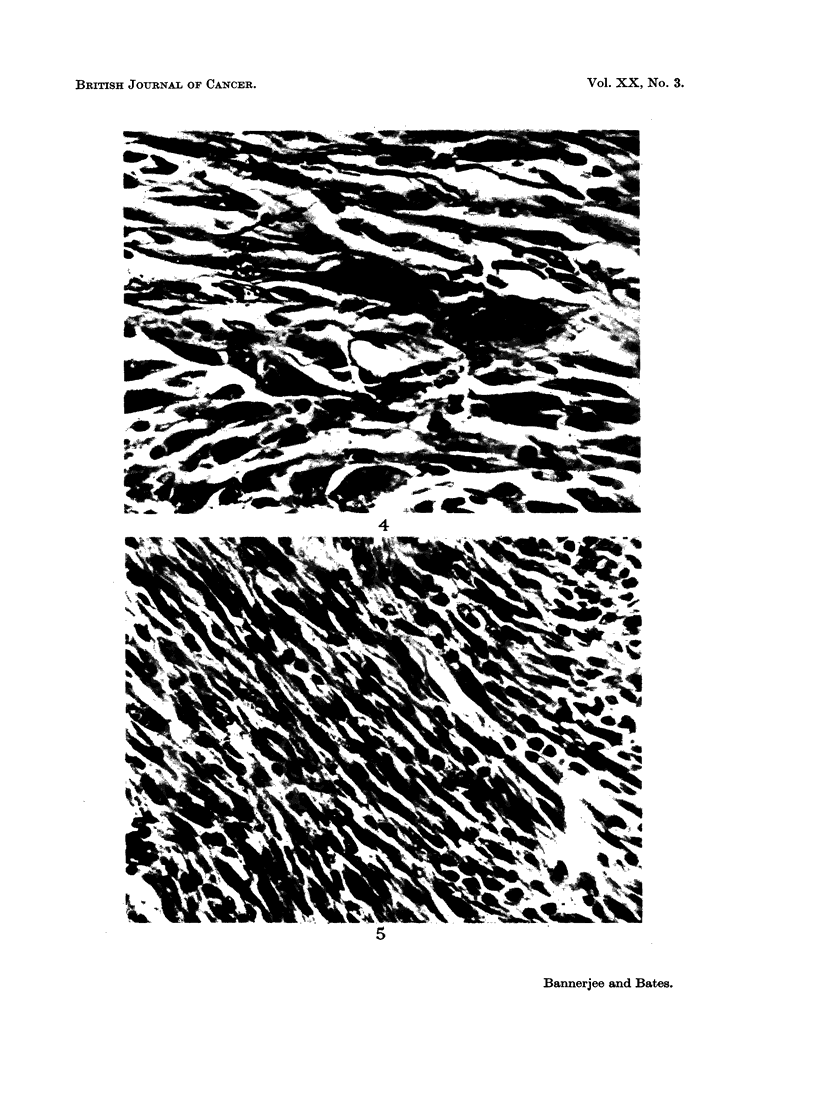

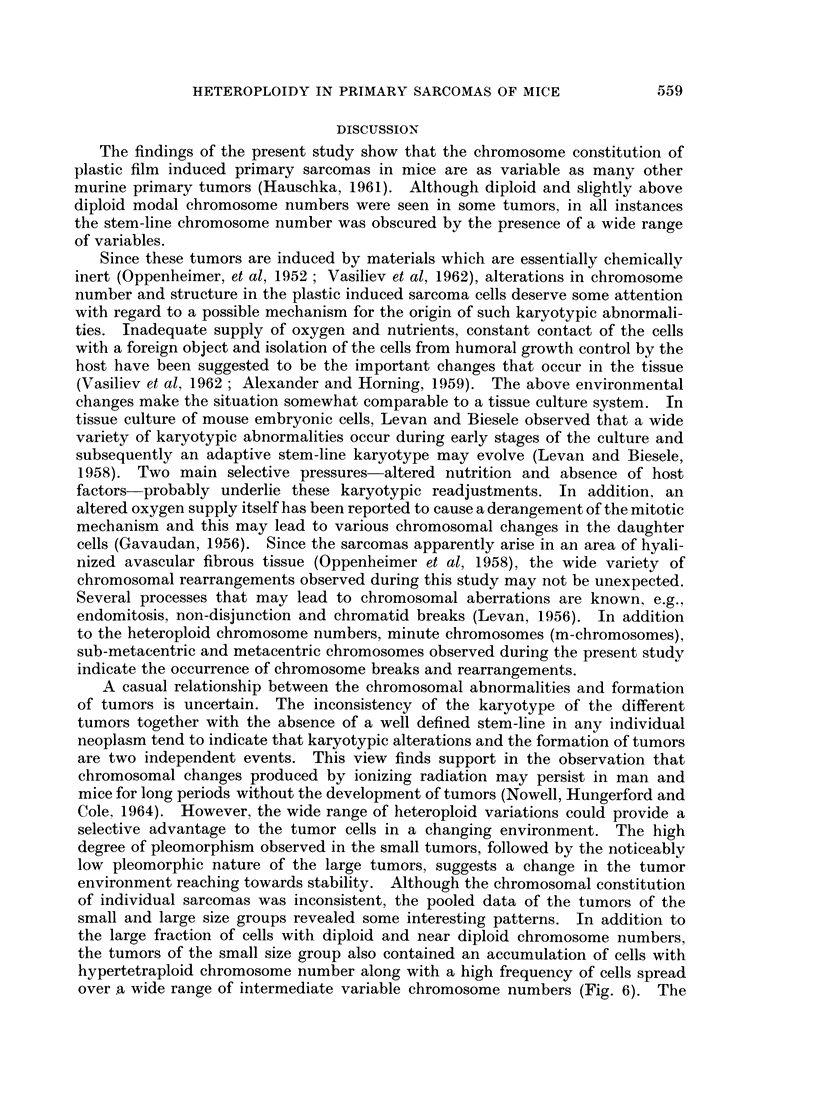

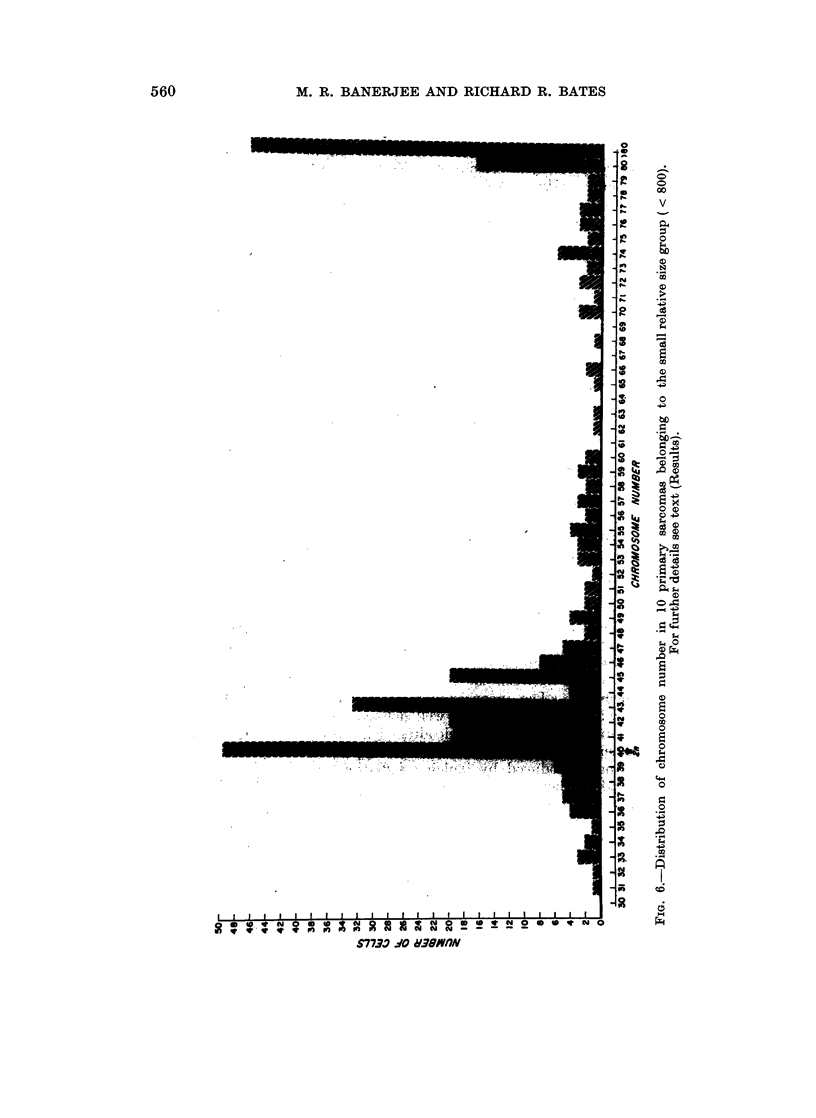

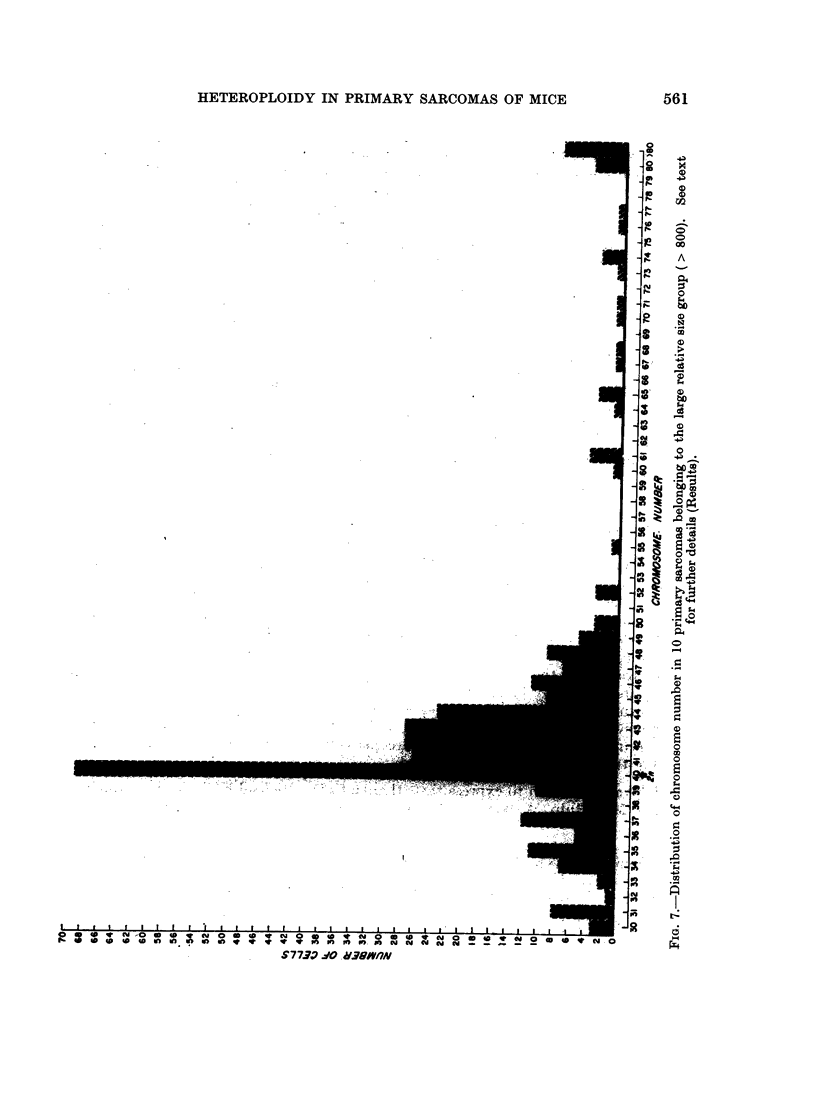

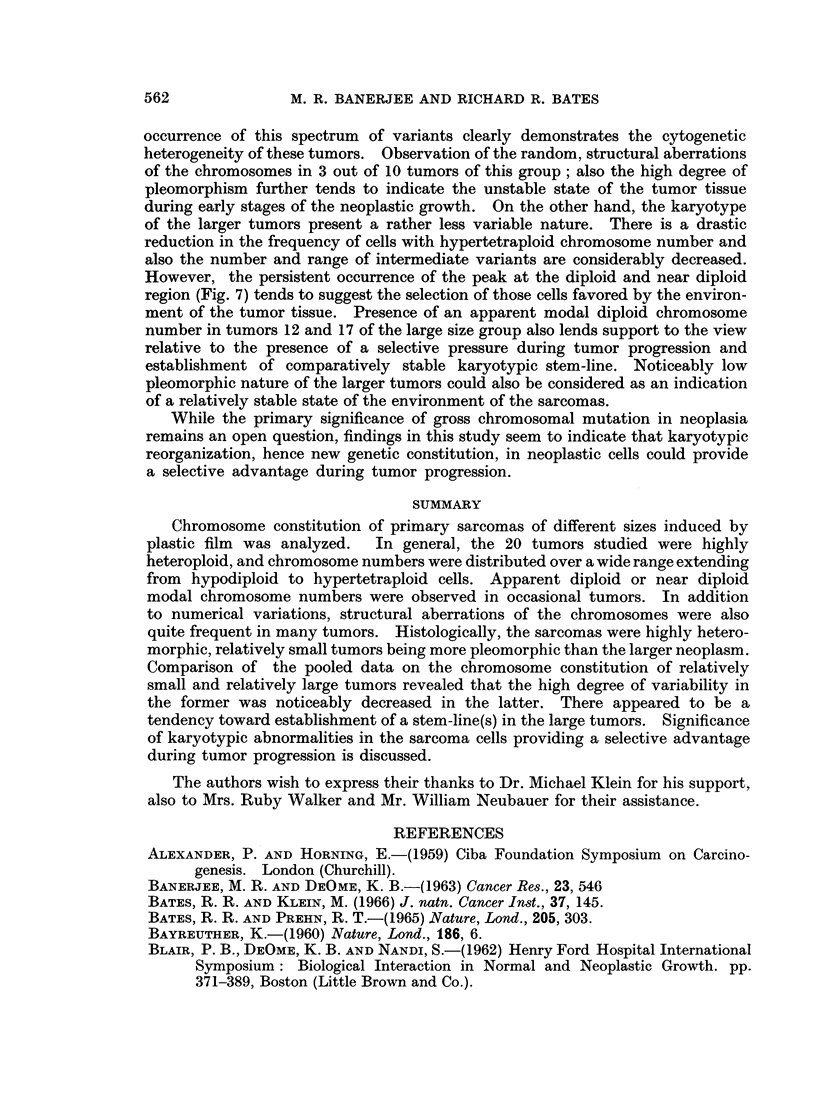

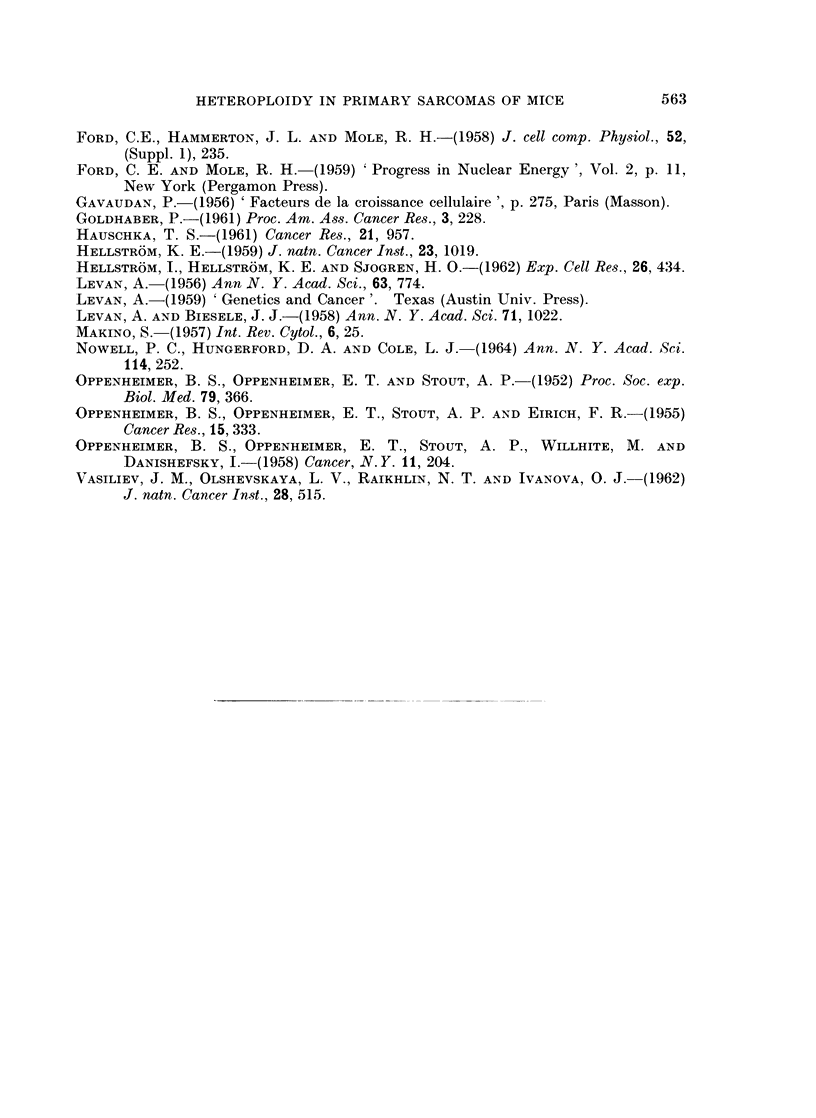


## References

[OCR_00560] BANERJEE M. R., DEOME K. B. (1963). Chromosomes in normal, preneoplastics, and neoplastic tissues of the mammary glands of C3H/Crgl female mice.. Cancer Res.

[OCR_00562] BATES R. R., PREHN R. T. (1965). ROLE OF THE FIBROUS CAPSULE IN CARCINOGENESIS BY PLASTIC FILM.. Nature.

[OCR_00561] Bates R. R., Klein M. (1966). Importance of a smooth surface in carcinogenesis by plastic film.. J Natl Cancer Inst.

[OCR_00582] HAUSCHKA T. S. (1961). The chromosomes in ontogeny and oncogeny.. Cancer Res.

[OCR_00586] HELLSTROM K. E., HELLSTROM I., SJOGREN H. O. (1962). Karyologic studies on polyoma virus induced mouse tumors.. Exp Cell Res.

[OCR_00592] LEVAN A., BIESELE J. J. (1958). Role of chromosomes in cancerogenesis, as studied in serial tissue culture of mammalian cells.. Ann N Y Acad Sci.

[OCR_00588] LEVAN A. (1956). Chromosomes in cancer tissue.. Ann N Y Acad Sci.

[OCR_00594] NOWELL P. C., HUNGERFORD D. A. (1964). CHROMOSOME CHANGES FOLLOWING IRRADIATION IN MAMMALS.. Ann N Y Acad Sci.

[OCR_00602] OPPENHEIMER B. S., OPPENHEIMER E. T., DANISHEFSKY I., STOUT A. P., EIRICH F. R. (1955). Further studies of polymers as carcinogenic agents in animals.. Cancer Res.

[OCR_00598] OPPENHEIMER B. S., OPPENHEIMER E. T., STOUT A. P. (1952). Sarcomas induced in rodents by imbedding various plastic films.. Proc Soc Exp Biol Med.

[OCR_00606] OPPENHEIMER B. S., OPPENHEIMER E. T., STOUT A. P., WILLHITE M., DANISHEFSKY I. (1958). The latent period in carcinogenesis by plastics in rats and its relation to the presarcomatous stage.. Cancer.

[OCR_00610] VASILIEV J. M., OLSHEVSKAJA L. V., RAIKHLIN N. T., IVANOVA O. J. (1962). Comparative study of alterations induced by 7,12-dimethylbenz[a]anthracene and polymer films in the subcutaneous connective tissue of rats.. J Natl Cancer Inst.

